# Supplementary material for: A microfluidic platform for extraction and analysis of bacterial genomic DNA
Source: Lab Chip. 2025 Feb 25;25(7):1767–75. doi: 10.1039/d4lc00839a (PMC11873781; doi:10.1039/d4lc00839a)
Supplement: LC-025-D4LC00839A-s001 [file LC-025-D4LC00839A-s001.pdf]

Supplementary Information (SI) for Lab on a Chip.
This journal is © The Royal Society of Chemistry 2025

Supporting information

A microfluidic platform for extraction and analysis of bacterial genomic DNA

Alex Joesaar, Martin Holub, Leander Lutze, Marco Emanuele, Jacob Kerssemakers, Martin Pabst, and Cees Dekker

Delft University of Technology, Van der Maasweg 9, 2629 HZ Delft, the Netherlands

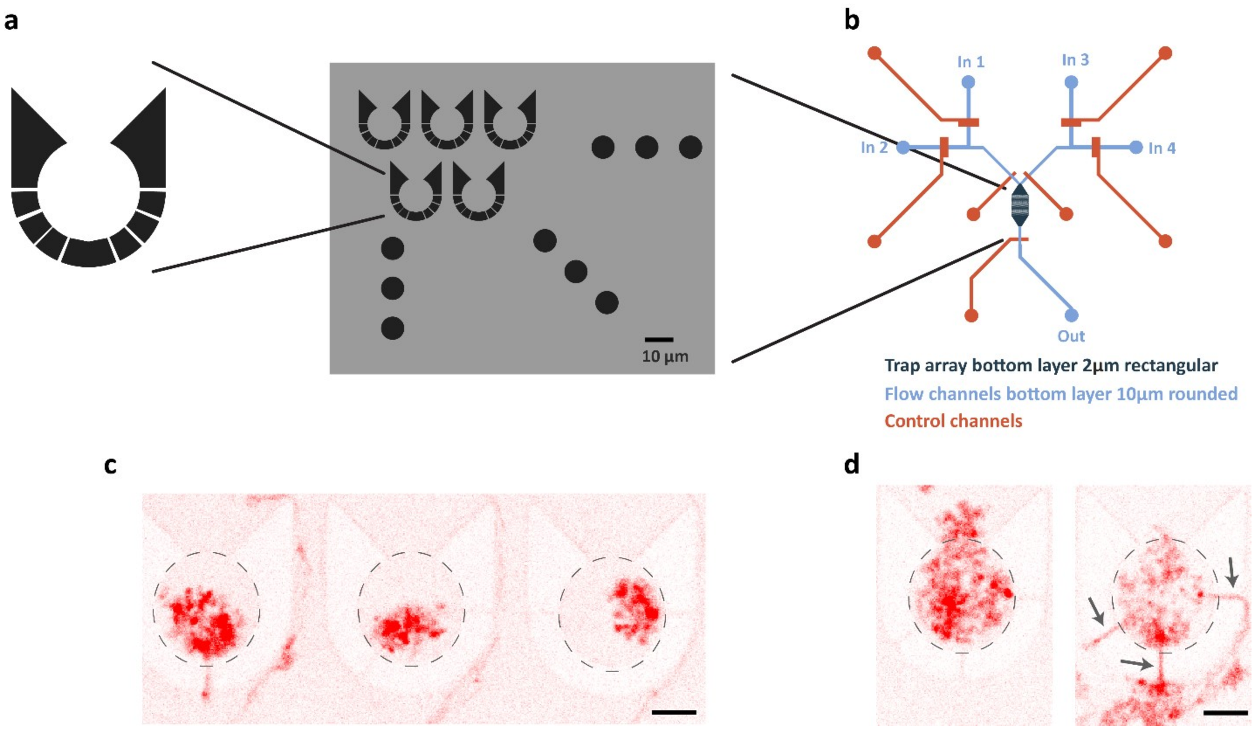

Supplementary Figure 1. Initial design of the microfluidic trapping device for bacterial chromosome extraction. **a**. Configuration of a flow cell with a 2D grid of PDMS traps. **b**. Schematic of the complete microfluidic chip. The left inputs (in1 and in2) were used for loading the cells while the right inputs (in3 and in4) were used for loading the lysis buffer. **c**. Confocal fluorescence micrograph of extracted genomic DNA from 3 *B. subtilis* cells labeled with Sytox Orange. Scale bar is 5 µm. **d**. An example of DNA leakage through the narrow slits of the PDMS trap. Scale bar is 5 µm.

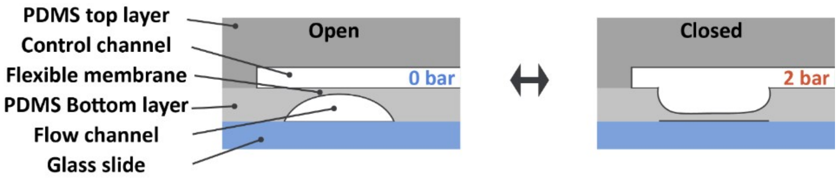

Supplementary Figure 2. Design and fabrication of pneumatically actuated microfluidic valves. Push-down configuration was used for all the valves. The rounded profile in the mold for valves 1, 2, 3, and 4 was realized by reflowing AZ10XT photoresist at 120C. All the control channels are 20 μm high; the flexible PDMS membrane has a thickness of approximately 15 μm; the flow channels are approximately 10 μm high in the center.

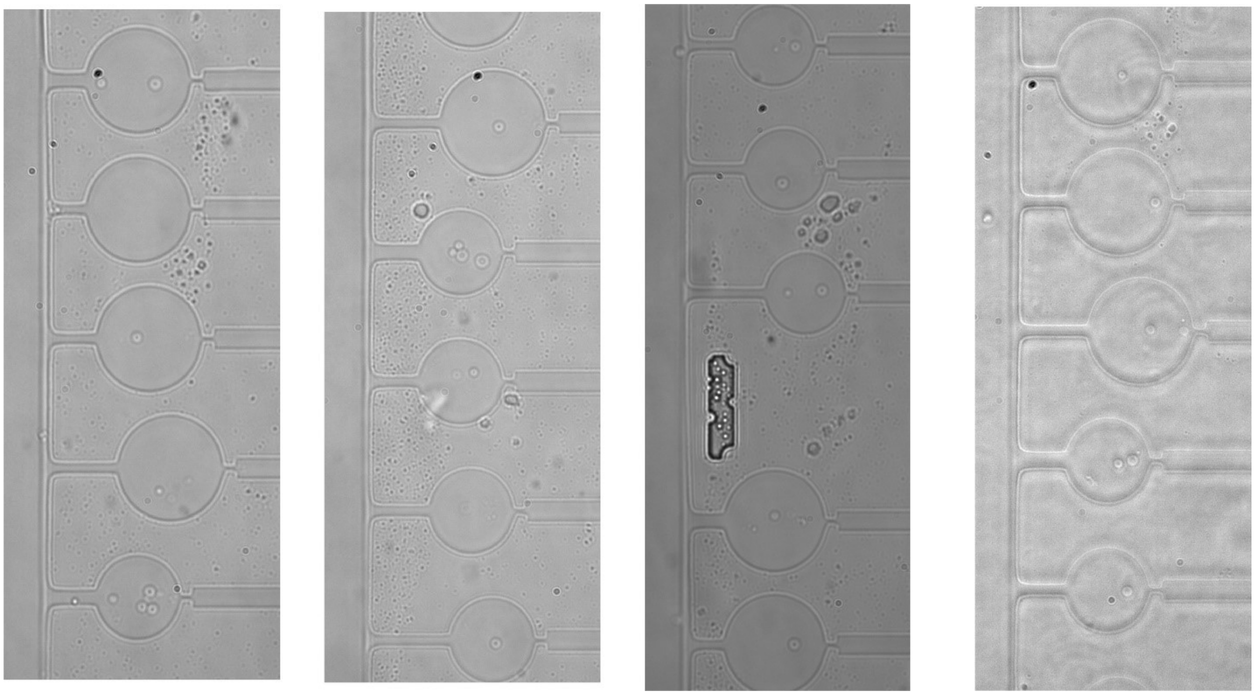

Supplementary Figure 3. Widefield micrographs of *B. subtilis* spheroplasts in the microfluidic trapping chambers. Approximately 40% of the traps contain a single spheroplast. The length of the input channels to the traps was varied for optimization purposes. Longer input channels resulted in a more robust trapping. When reagents were flowed through the flow channel, however, longer channels also increased the time that it took for reagents to diffuse into the traps.

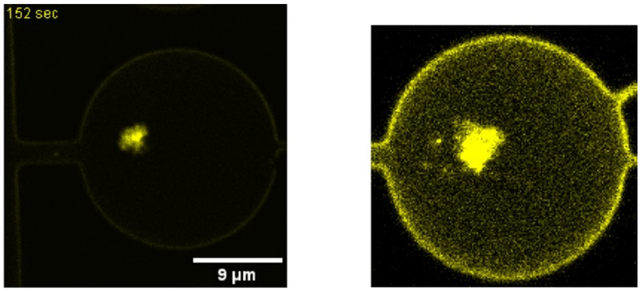

Supplementary Figure 4. Lysis results using osmotic shock. Two examples of Sytox-Orange-labeled genomic DNA that was extracted using an osmotic shock, resulting in a more compacted structure.

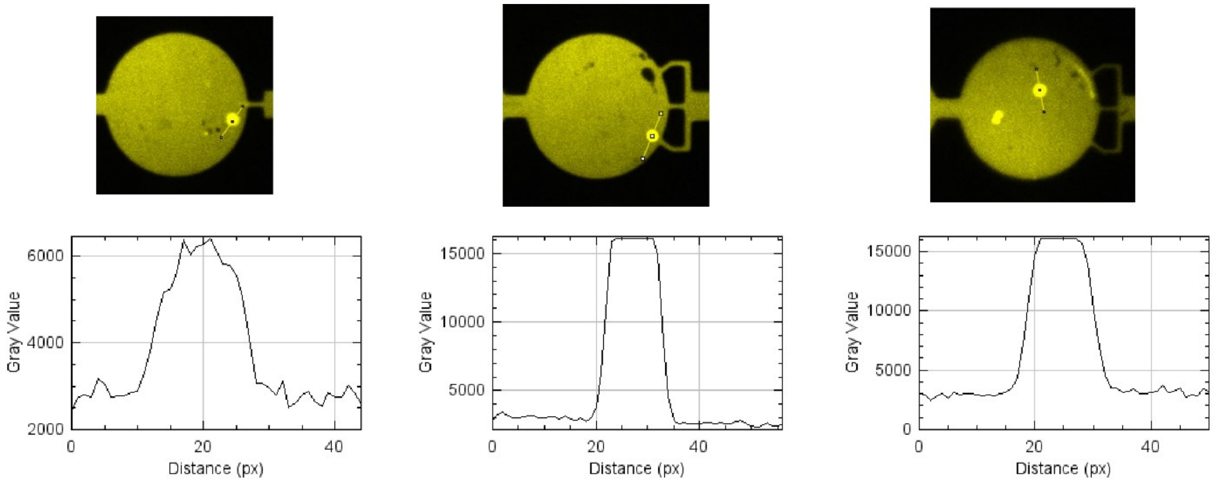

Supplementary Figure 5. Signal-to-background ratio of Alexa647-NHS protein quantification measurements. Three examples of Alexa647 fluorescence intensity profiles of spheroplasts before lysis.

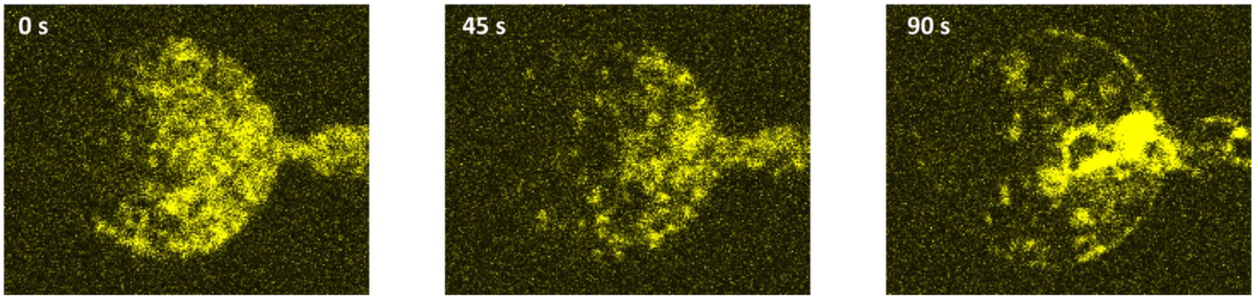

Supplementary Figure 6. Adsorption of condensed DNA to chamber walls. An example of DNA condensation and absorption to the bottom of the trapping chamber upon addition of 20% PEG solution.

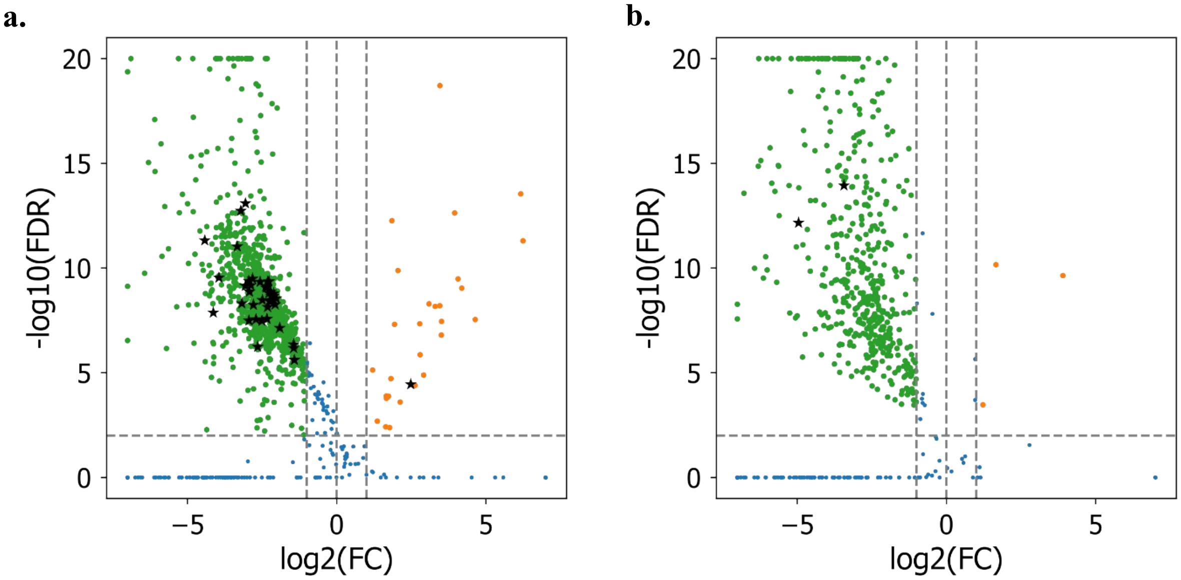

Supplementary Figure 7: Protein abundance after removal for **a**. *E. coli* **b**. *B. subtilis*. Vertical lines indicate 2-fold removal and enrichment respectively, horizontal line corresponds to significance threshold of 1%. Points highlighted with stars represent DNA-binding proteins (cf. Table S2 and S3).

	E. coli	*B. subtilis*
**Relative abundance [%]**		
DNA-binding	19.2 ± 6.6	9.9 ± 2.6
All	24.6 ± 8.5	17.7 ± 4.4
**Number of proteins reduced fewer than two-fold**		
DNA-binding	1‡ (of 39)	0 (of 2)
All	69 (of 1246)	13 (of 490)

Table S1: Relative protein abundance after treatment for *E. coli* and *B. subtilis,* and the number of proteins that were not reduced more than 2-fold. Proteins were selected on significance threshold of 1% on the fold-change. To obtain relative values, proteins were weighted by their mass. ‡ RpoZ

Protein Name	Description
hbs	DNA-binding protein HU 1
rpoY	DNA-directed RNA polymerase subunit epsilon

Table S2: Proteins labeled as DNA-binding or DNA-processing in the *B. subtilis* sample (filtered on FDR 1%).

Protein Name	Description
dnaE	DNA polymerase III subunit alpha
topA	DNA topoisomerase 1
cbpA	Curved DNA-binding protein
dps	DNA protection during starvation protein
matP	Macrodomain Ter protein
gyrA	DNA gyrase subunit A
mukE	Chromosome partition protein MukE
rpoB	DNA-directed RNA polymerase subunit beta
ybaB	Nucleoid-associated protein YbaB
hupA	DNA-binding protein HU-alpha
parC	DNA topoisomerase 4 subunit A
mukF	Chromosome partition protein MukF
ybiB	Uncharacterized protein YbiB
hupB	DNA-binding protein HU-beta
mukB	Chromosome partition protein MukB
crp	DNA-binding transcriptional dual regulator CRP
rpoC	DNA-directed RNA polymerase subunit beta'
ompR	DNA-binding dual transcriptional regulator OmpR
ihfB	Integration host factor subunit beta
rpoA	DNA-directed RNA polymerase subunit alpha
rpoD	RNA polymerase sigma factor RpoD
crl	Sigma factor-binding protein Crl
dnaN	Beta sliding clamp
ihfA	Integration host factor subunit alpha
polA	DNA polymerase I
fis	DNA-binding protein Fis
uvrA	UvrABC system protein A
gyrB	DNA gyrase subunit B
nadR	Trifunctional NAD biosynthesis/regulator protein NadR
rpoS	RNA polymerase sigma factor RpoS
yaaA	DNA-binding and peroxide stress resistance protein YaaA
uvrD	DNA helicase II
yejK	Nucleoid-associated protein YejK
oxyR	DNA-binding transcriptional dual regulator OxyR
stpA	DNA-binding protein StpA
parE	DNA topoisomerase 4 subunit B
hns	DNA-binding protein H-NS
kdgR	HTH-type transcriptional regulator KdgR
rpoZ	DNA-directed RNA polymerase subunit omega

Table S3: Proteins labeled as DNA-binding or DNA-processing in the *E. coli* sample (filtered on FDR 1%).
